# Comparative Proteome Analysis of Porcine Jejunum Tissues in Response to a Virulent Strain of Porcine Epidemic Diarrhea Virus and Its Attenuated Strain

**DOI:** 10.3390/v8120323

**Published:** 2016-11-29

**Authors:** Zhonghua Li, Fangzhou Chen, Shiyi Ye, Xiaozhen Guo, Atta Muhanmmad Memon, Meizhou Wu, Qigai He

**Affiliations:** State Key Laboratory of Agricultural Microbiology, College of Veterinary Medicine, Huazhong Agricultural University, Wuhan 430070, China; lzh1990@webmail.hzau.edu.cn (Z.L.); chenfangzhou@webmail.hzau.edu.cn (F.C.); yeshiyi@webmail.hzau.edu.cn (S.Y.); gxz0821@163.com (X.G.); Memonatta80@webmail.hzau.edu.cn (A.M.M.); wumeizhou@mail.hzau.edu.cn (M.W.)

**Keywords:** virulent, attenuated, porcine epidemic diarrhea virus, PEDV, quantitative proteome analysis, iTRAQ

## Abstract

Porcine epidemic diarrhea virus (PEDV), a predominant cause of acute enteric infection, leads to severe dehydrating diarrhea and mortality in piglets all over the world. A virulent PEDV YN13 strain, isolated in our laboratory, was attenuated to yield an attenuated PEDV strain YN144. To better understand the pathogenesis mechanism and the virus-host interaction during infection with both PEDV YN13 and YN144 strains, a comparative proteomic analysis was carried out to investigate the proteomic changes produced in the primary target organ, using isobaric tags for relative and absolute quantitation (iTRAQ) labeling, followed by liquid chromatography tandem-mass spectrometry (LC-MS/MS). A total of 269 and 301 differently expressed proteins (DEPs) were identified in the jejunum tissues of the piglets inoculated with YN13 and YN144, respectively. Bioinformatics analysis revealed that these proteins were involved in stress responses, signal transduction, and the immune system. All of these involved interferon-stimulated genes (ISGs) which were up-regulated in jejunums by both of the PEDV-infected groups. Based on the comparative analysis, we proposed that different changes induced by YN13 and YN144 in heterogeneous nuclear ribonucleoprotein A1 (hnRNPA1), eukaryotic initiation factor 4G1 (eIF4G1), and some members in the heat shock protein (HSP) family, may be responsible for differences in their pathogenicity.

## 1. Introduction

Porcine epidemic diarrhea (PED), an acute enteric disease of pigs, is characterized by vomiting, watery diarrhea, dehydration, and high mortality in piglets [1]. PED was first reported in England in 1971 [2]. Since then, it has been reported in many European and Asian countries. In the past ten years, new outbreaks of PED in Thailand [3], China [4,5,6], South Korea [7,8], USA [9], and Japan [10] have caused serious economic losses to their swine industry. For example, the PED outbreak in USA in 2013 led to an estimated death of 7 million piglets [11]. The etiological agent is porcine epidemic diarrhea virus (PEDV). PEDV, a member of the *Coronaviridae* family, is a single-stranded positive sense RNA virus. Viral genome is about 28 kb, encoding four structural proteins (spike (S), membrane (M), envelope (E), and nucleocapsid (N)), and three non-structural proteins (replicases 1a, 1b, and accessory protein ORF3) [12]. Despite extensive research on PEDV, there have been few comparative in vivo studies examining the host interaction with the wild type, its attenuated PEDV strain, and their pathogenesis.

Due to the advantages of convenience and high sensitivity, isobaric tags for relative and absolute quantitation (iTRAQ)-based quantitative proteomic technology is considered an excellent option to analyze host-viral interactions. In recent years, this technology has been widely used to investigate the alterations in cellular proteins during infection by many viruses, such as human immunodeficiency virus (HIV) [13], influenza virus [14,15], transmissible gastroenteritis virus (TGEV) [16,17], as well as PEDV [18,19,20]. To the best of our knowledge, all the three recently published studies on proteomic changes in host cells infected with PEDV have been performed in vitro using Vero cells. However, the in vivo infection model may provide more biologically relevant insights into pathogenesis.

The small intestine, especially the jejunum, has been reported as the target tissue of PEDV infection [21]. Therefore, in the present study, iTRAQ labeling coupled with liquid chromatography tandem-mass spectrometry (LC-MS/MS) was used to analyze whole cell changes in jejunum of piglets infected with a highly virulent PEDV strain YN13 and its cell-adapted strain YN144 for a better understanding of the virus-host interaction during PEDV infection and variation in the pathogenicity of these two strains.

## 2. Materials and Methods

### 2.1. Animal and Virus Strains

Twelve piglets were purchased from a farm without any history of PED. The antibody free to PED was confirmed by a PEDV ELISA Kit (Shanghai Shifeng Biological Technology Co., Ltd., Shanghai, China). The absence of PEDV was confirmed by real time PCR (RT-PCR) based on the M gene of PEDV. The virulent strain, YN13, (Accession No. KT021228) was isolated from the intestine of a piglet with diarrhea and passaged for 13 generations, and the attenuated strain, YN144 (Accession No. KT021232), was obtained by further passaging of YN13 for 144 generations. The attenuation method and genomic comparison were reported in our previous study [22]. Briefly, the virulent variant of PEDV strain YN-1 was serially passaged in the Vero cells with serum-free Dulbecco's Modified Eagle Medium (DMEM) containing 8 μg/mL trypsin (Invitrogen, Carlsbad, CA, USA) for attenuation. The genomic characteristics of the parental strain YN1 and different passages, in other words, YN15, YN30, YN60, YN90, YN144, and YN200, were compared at both the nucleotide and protein level.

### 2.2. Experimental Design

Twelve seven-day-old piglets were randomly divided into three groups: the YN13-infected group, YN144-infected group, and the control group, with each group consisting of four piglets and housed in a different piggery. After three days of acclimation, piglets of the corresponding groups were orally inoculated with 4.22 mL of PEDV YN13 and YN144 strains at a titer of 10^5.375^ median tissue culture infective dose (TCID50) mL^−1^. The piglets in the control group were mock-inoculated in parallel with 4.22 mL of sterile DMEM.

All piglets were euthanized and necropsied when diarrhea was observed in the piglets of the YN13-infected group. Proximal jejunum tissues were separated rapidly, washed with ice-cold phosphate-buffered saline (PBS) buffer, snap-frozen in liquid nitrogen, and kept at −80 °C for subsequent proteome analysis. Proximal jejunum samples were also collected for quantitative PEDV detection. Parenchymal organs and each part of gastrointestinal tract were collected and then fixed in 10% neutral buffered formalin for histopathological examination and immunohistochemistry (IHC) analysis.

### 2.3. Histopathology and IHC Analysis

Tissue samples collected from parenchymal organs and the gastrointestinal tract were fixed in 10% neutral buffered formalin, and routinely processed into paraffin. For pathological examination, sections of different tissues of 2–3 μm were cut for hematoxylin and eosin (H&E) staining. Villus height and crypt depth were measured by moditec photographic processing software and a pathological image analysis system. Statistical analysis of villus height and crypt depth ratio was performed using SPSS statistics 17.0. For immunohistochemistry analysis, sections of about 4 μm thickness were prepared, and microwave treated in 10 mM sodium citrate solution (pH 6.0) for antigen retrieval. Then, 5% bovine serum albumin (BSA) blocking buffer was used to block non-special binding. After blocking, anti-PEDV monoclonal antibodies (Jeno Biotech Inc., Chuncheon, Korea) at a dilution of 1:100 were applied to detect the spike protein of PEDV and allowed to incubate overnight at 4 °C. Biotinylated goat anti-mouse IgG (BOSTER, Wuhan, China) was used to detect the primary antibody. The antigen-antibody complexes were detected with streptavidin-biotin complex (SABC) kit (BOSTER, Wuhan, China). Diaminobenzidine (DAB) and hematoxylin served as the substrate chromogen and counterstain, respectively. Immunohistochemical images were captured digitally. The mean density (integrated optical density sum/ positive area sum) of the DAB-stained areas in each photo as measured by Image-Pro Plus 6.0 software (Media Cybernetics, Rockville, MD, USA).

### 2.4. Quantitative Analysis of PEDV in Jejunum by Real-Time RT-PCR

A total of 100 mg of jejunum homogenates were suspended in 500 µL PBS, freeze-thawed three times at −80 °C, and centrifuged at 12,000× *g*, for 10 min, at 4 °C. Next, 200 μL of clarified supernatant was transferred into a separate sterile tube for total RNA extraction by using Tripure Isolation Reagent (Roche, Indianapolis, USA) according to the manufacturer’s instructions. The cDNA was obtained by RT-PCR using the PrimescriptTM RT Master Mix (TakaRa, Tokyo, Japan). The real-time RT-PCR assay used the following primer and probe sequences: PEDV forward primer: 5′-CGTACAGGTAAGTCAATTAC-3′, PEDV reverse primer: 5′-GATGAAGCATTGACTGAA-3′, and PEDV Taq-Man^®^probe: FAM-TTCGTCACAGTCGCCAAGG-TAMRA. The PCR was performed using the following parameters: 50 °C for 2 min, 95 °C for 10 min, 40 cycles with 95 °C for 15 s, 56 °C for 30 s and 72 °C for 31 s.

### 2.5. Protein Extraction, Digestion, and Labeling with iTRAQ Reagents

Jejunum samples were treated with 150 µL radioimmunoprecipitation assay (RIPA) buffer (50 mM Tris-Hcl, 150 Mm NaCl, 1% SDS, 0.1% Trionx-100, 1% SDC, pH 8.0) containing 1 mM phenylmethane sulfonyl fluoride (PMSF). Next, protein solubilization was achieved by sonication, and cellular debris was removed by centrifugation (12,000× *g*, 20 min, 4 °C). The protein concentration in the middle layer of the liquid was quantified by Bradford protein assay. Then, equal portions of three independent biological replicates in each group were mixed to make one sample according to the results of the Bradford protein assay. A total of 200 μg of sample was digested and then aliquoted to two equal parts. The protein samples from the control group, YN144-infected group, and YN13-infected group were labeled with iTRAQ 113 or iTRAQ 114, iTRAQ 115 or iTRAQ 116, and iTRAQ 119 or iTRAQ 121, respectively. The labeled digests were then pooled and vacuum-dried.

### 2.6. High-pH Reversed-Phase Chromatography

These iTRAQ labeled peptide samples were then redissolved in 200 µL buffer A solution (20 mM HCOONH4, 2 M NaOH, pH 10). The dissolved sample (100 µL) was passed through high-performance liquid chromatography (HPLC) on Phenomenex columns (Gemini-NX 3u C18 110 A; 150*2.00 mm). Before HPLC, Phenomenex columns were balanced with 95% buffer A and 5% buffer B (20 mM HCOONH4, 2 M NaOH, 80% acrylonitrile [CAN], pH 10), for 30 min. Peptides were separated by a linear gradient formed from buffer A and buffer B, from 5% to 50% of buffer B at a flow rate of 0.2 mL/min. The ultraviolet (UV) detector was set at 214 nm and a total of 24 fractions were collected.

### 2.7. RPLC-MS Analysis

Peptide fractions collected through HPLC were dissolved in formic acid (FA)-acrylonitrile (ACN) solution (2% ACN, 0.1% FA), thoroughly vortexed, and finally centrifuged at 13,200× *g* for 20 min at 4 °C. Peptides were separated by a linear gradient formed from mobile phase A (0.1% FA, 5% ACN) and mobile phase B (0.1% FA, 95% ACN), from 5% to 40% of mobile phase B for 70 min, at a flow rate of 300 nL/min. For MS analysis, a TripleTOF 5600 system (AB SCIEX, Foster City, CA, USA) was used in Information Dependent Mode. MS spectra were acquired across the range of 400–1250 *m*/*z* in high resolution mode (>30,000), using 250 ms accumulation time per spectrum. A maximum of 20 precursors per cycle were chosen for fragmentation from each MS spectrum with 100 ms minimum accumulation time for each precursor and dynamic exclusion for 20 s. Tandem mass spectra were recorded in high sensitivity mode (resolution > 15,000), with turned on rolling collision and iTRAQ reagent collision energy adjustment.

### 2.8. Data Analysis

The MS raw data files were converted to mascot generic format (MGF) files by ProteinPilot™ Software 4.5 (AB SCIEX, Foster City, CA, USA). MS/MS data were searched against the *Sus scrofa* subset database from the UniProt database. The paragon algorithm (AB SCIEX) was applied for peptide identification and quantification. The parameters for data search were set up as follows: trypsin (KR) cleavage with two missed cleavage sites was considered along with fixed modification of cysteines by methyl methanethiosulfonate (MMTS). iTRAQ modification of peptide/protein identification and quantification was also performed with the ProteinPilot software version 4.5 (AB SCIEX, Foster City, CA, USA). The database and data search parameters remained the same as above. A strict cutoff for protein identification was applied with an unused ProtScore ≥1.3, which corresponded to a confidence limit of 95%, to minimize false positive results. At least two peptides with 95% confidence were considered for protein quantification.

The peptide used in iTRAQ quantification was automatically selected by the pro group algorithm to calculate the reported peak area, error factor (EF), and *p*-value. The resulting data set was auto bias-corrected to eliminate any variation due to unequal mixing when different labeled samples were combined. False discovery rate (FDR) analysis was also performed using the integrated tools in ProteinPilot (FDR < 0.01). For data analysis, protein quantification data with a fold change of >2 and <0.5 and *p*-value < 0.05 was selected as the significantly differently expressed protein (DEP). Bioinformatics analyses of these DEPs were conducted using Ingenuity Pathway Analysis (IPA) software (QIAGEN, Dusseldorf, Germany), GO, and UniProt.

### 2.9. Western Blot Analysis

Aliquots of sample lysates were subjected to sodium dodecyl sulfate polyacrylamide gel electrophoresis (SDS-PAGE), followed by transferring proteins to polyvinylidene difluoride (PVDF) membrane. The membrane was blocked with 5% (*w*/*v*) skim milk in Tris-buffered saline (TBS), containing 0.05% Tween-20 (TBST), at 37 **°**C for 2 h, followed by washing three times with TBST. Then the membrane was incubated overnight at 4 °C with mouse polyclonal antibody against β-actin (ABclonal, Wuhan, China), rabbit polyclonal antibodies against HSP90B1, ANXN4, APN (ABclonal), or MX1, (Protein-Tech, Wuhan, China). After washing three times with PBST, the membrane was incubated with Horseradish peroxidase (HRP)-conjugated goat anti-mouse/rabbit IgG (ABclonal) at 37 °C for 2 h. Finally, the protein bands were visualized using the Clarity™ Western ECL Blotting Substrate (Bio-Rad, Hercules, CA, USA).

## 3. Result

### 3.1. Clinical Observations and Pathological Examination

Experimental infection in piglets with YN144 strain did not exhibit any symptoms of vomiting and diarrhea, while all the piglets in the YN13-infected group showed apparent diarrhea at 3 days post infection (dpi) (Supplementary Materials Figure S1). All piglets were euthanized and necropsied at 3 dpi.

**Figure 1:**
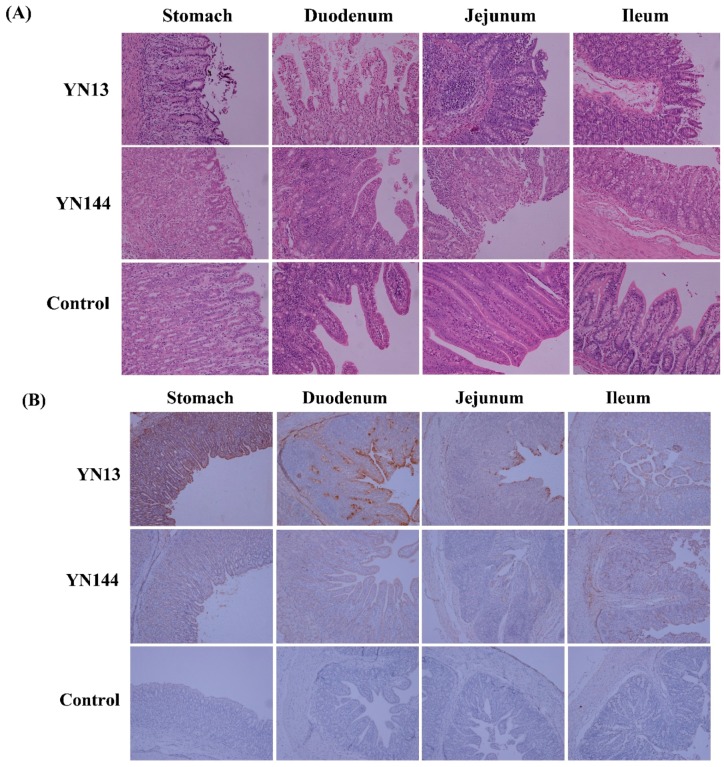

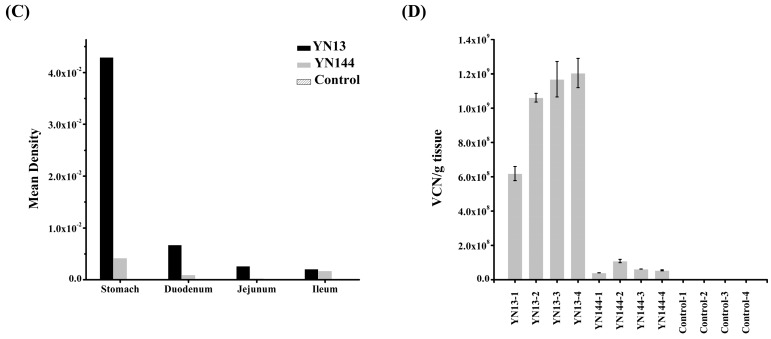
Comparison of YN13 and YN144 infection in target tissues. (**A**) Histopathological analysis of the YN13-infected, YN144-infected, and control groups (**B**) Immunohistochemical assay of the YN13-infected, YN144-infected, and control groups; (**C**) Mean density of porcine epidemic diarrhea virus (PEDV) in each tissue shown in (**B**); (**D**) The viral load in jejunum sample of each piglet was quantified using real-time RT-PCR. The average viral copy number (VCN) per g tissue of each piglet was calculated. Error bars indicate standard error from the mean, and the horizontal axis indicates the number of piglets.

Serious pathological damage was found in the stomach of the YN13 infected group, including the shortening and coarsening of villi, and the desquamation of epithelial cells (Figure 1A). Pathological changes were found in the small intestine of the virus-infected groups, including the irregularity and desquamation of epithelial cells, the defected and irregular striated border, the shortening and coarsening of villi and the increase of goblet cells (Figure 1A). However, lesions in the small intestine of piglets in the YN13-infected group were more serious than those in the YN144-infected group, demonstrating that YN13 had stronger pathogenicity than YN144. Villous height and crypt depth ratio (V/C) was also measured to show the stronger pathogenicity of YN13 than YN144 (Supplementary Materials Table S1). Villus height and crypt depth ratios of jejunum and ileum in the YN13-infected group were less than those in the YN144-infected group (*p* < 0.01) (Supplementary Materials Figure S2).

### 3.2. Immunohistochemistry and Virus Detection in Jejunum

Immunohistochemistry was carried out to explore the presence of PEDV. PEDV could only be detected in the gastric mucosa, epithelium, gastric submucosa, intestinal epithelial cells, crypt epithelial cells, and intestinal glands of the small intestine (Figure 1B). Despite no differences in the distribution of these two viruses, the quantity of positive signals was different. For an obvious comparison of the positive signals in the same tissue developed by two strains in their corresponding groups, mean densities of PEDV in the tissues, as shown in Figure 1B, were calculated. For the same tissue, more positive signals were detected in the YN13-infected group than in the YN144-infected group (Figure 1C).

Real-time PCR was used to quantify the viral loads in jejunum samples of all piglets. PEDV could be detected from the jejunum samples of all pigs in PEDV-infected groups, but not in the control group, suggesting the successful infection of all piglets in the PEDV-infected groups. In addition, consistent with immunohistochemistry analysis, viral loads in jejunum samples of the YN13-infected group were much higher than those in the YN144-group (Figure 1D), implying that the replication ability of YN13 in jejunums is stronger than that of YN144.

### 3.3. Analysis of the Differentially Regulated Proteins

In total, 3847 and 3877 proteins were detected in the YN13-infected group and the YN144-infected group, respectively. Of these proteins, 131 were upregulated and 138 were downregulated significantly (fold ≥ 2 or ≤0.5) in the YN13-infected group, while 173 were upregulated and 128 were downregulated significantly (fold ≥ 2 or ≤0.5) in the YN144-infected group (Supplementary Materials File S1).

**Figure 2:**
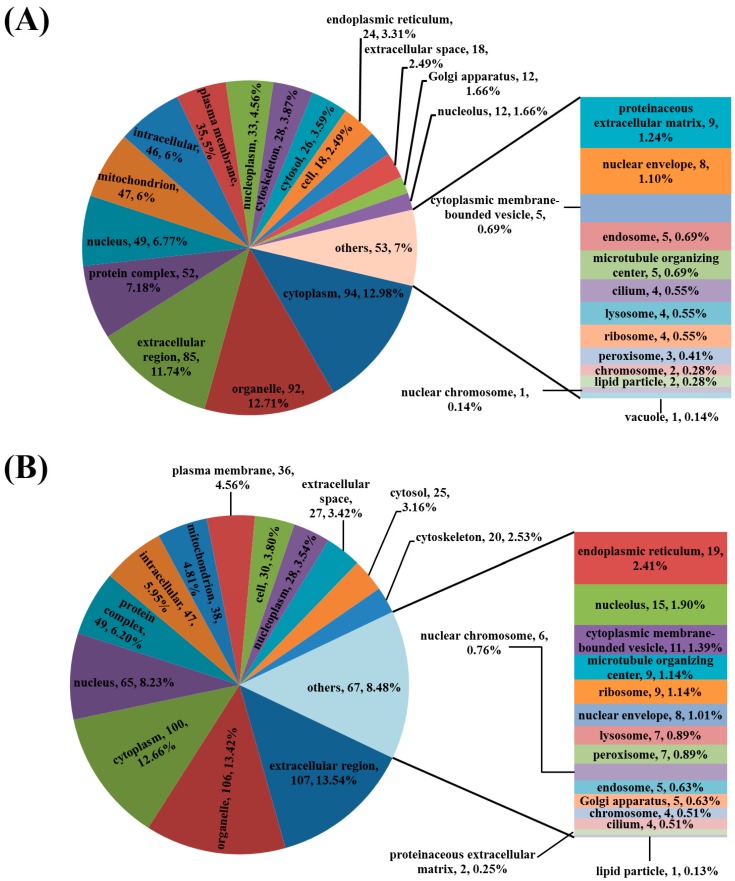
Subcellular location of the differently expressed proteins in jejunum of pigs infected with PEDV. (**A**) Differently expressed proteins in YN13-infected group; (**B**) Differently expressed proteins in YN144-infected group.

Biological processes, molecular functions and cellular components of all these proteins were analyzed by UniProt and GO database (Supplementary Materials File S2). As shown in Figure 2, DEPs in both of these two virus-infected groups were mainly localized in the cytoplasm, organelles, extracellular region, protein complexes, nuclei, mitochondria, and intracellular and plasma membranes.

**Figure 3:**
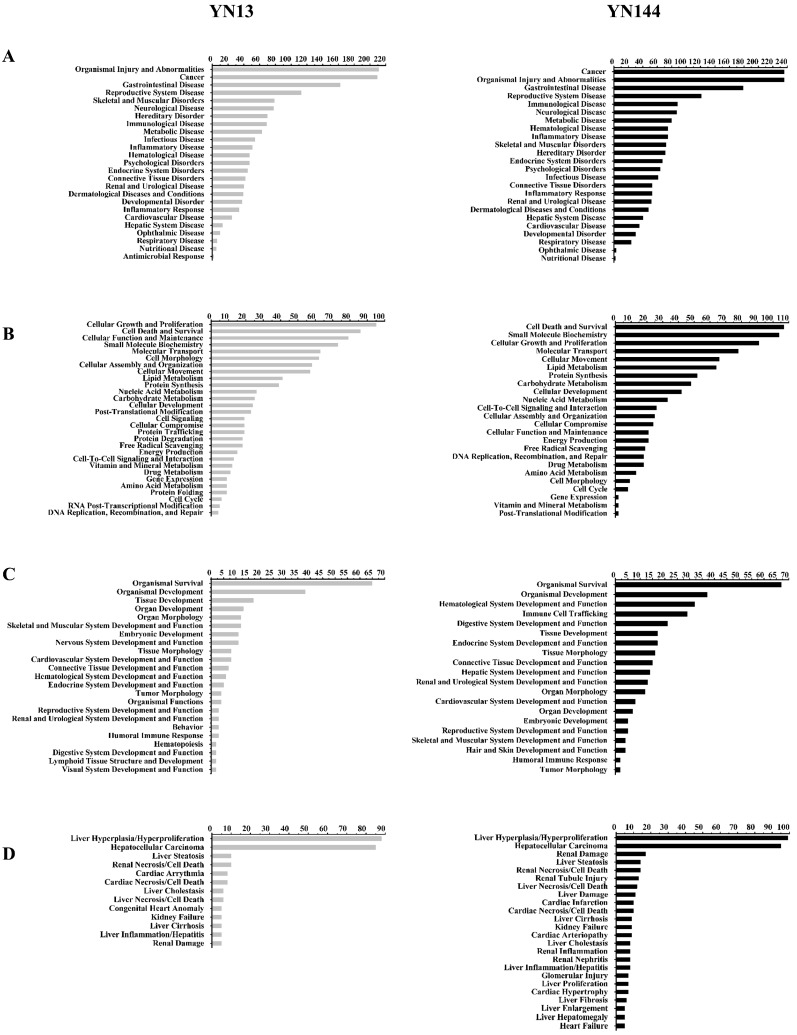
Functional characterization of significantly differently expressed proteins of YN13 and YN144-infected groups. (**A**) Diseases and disorders; (**B**) Molecular and cellular functions; (**C**) Physiological system development and functions; (**D**) Toxicity functions. More information is available in the Supplementary Materials File S3.

Because many proteins of pigs were not characterized, protein accession numbers in the Supplementary Materials File S1 were transformed to human protein accession numbers. Then, the protein accession numbers and fold changes were input into IPA software and the functional classification and biological processes of these proteins were constructed based on the underlying biological evidence from the literature. IPA analysis results for the two groups showed that DEPs could be divided into four functional classifications: diseases and disorders; molecular and cellular functions; physiological system development; and toxicity functions (Figure 3 and Supplementary Materials File S3). These differentially regulated proteins in the YN13 infected group and YN144 infected group were mainly involved in some common biological processes, including organismal injury abnormalities, gastrointestinal disease, skeletal and muscular disorders, infectious diseases, and cell death and survival, indicating that these two PEDV strains similarly affect host global response.

**Figure 4:**
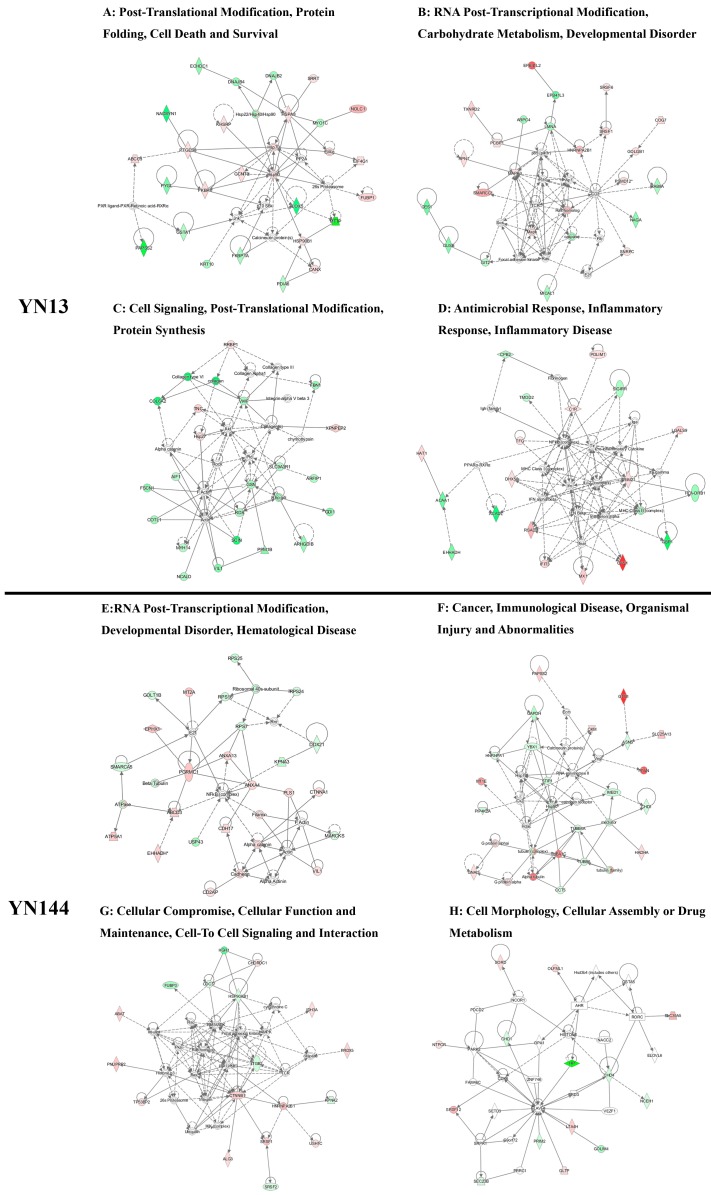
Specific network analysis of significantly differently expressed proteins for YN13 and YN144-infected groups. The red icons represent significantly upregulated proteins; the green icons represent significantly downregulated proteins; and the white icons represent proteins that were not identified in this study but are involved in the networks. The color shade indicates the magnitude of the change in protein expression. The shapes are indicative of the molecular class, and the lines with arrows indicate the molecular relationships (Supplementary Materials Figure S3).

Potential networks of DEPs were also conducted by the IPA tool. Networks of interest for the YN13-infected group corresponded to the following: (1) Post-translational modification, protein folding, cell death and survival (Figure 4A); (2) RNA post-transcriptional modification, carbohydrate metabolism (Figure 4B); (3) Cell signaling, post-translational modification, protein synthesis (Figure 4C); and (4) Antimicrobial response, inflammatory response, inflammatory disease (Figure 4D). Networks of interest for YN144-infected group corresponded to the following: (1) RNA post-transcriptional modification, developmental disorder, hematological disease (Figure 4E); (2) Cancer, immunological disease, organismal injury and abnormalities (Figure 4F); (3) Cellular comprise, cellular function and maintenance, cell-to-cell signaling and interaction (Figure 4G); and (4) Cell morphology, cellular assembly or drug metabolism (Figure 4H).

### 3.4. Validation of Protein Identification and Quantitation

**Figure 5:**
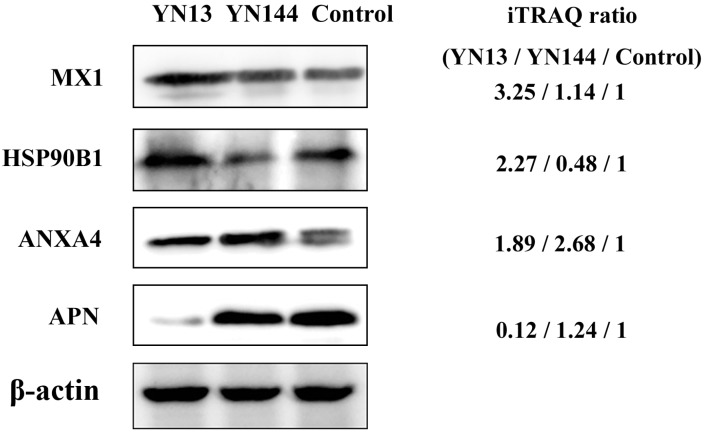
Confirmation of differentially expressed proteins by Western blot. Analysis of MX1, heat shock protein 90 kDa beta member 1 (HSP90B1), Annexin A4 (ANXA4), and Aminopeptidase N (APN) expression level in YN13-infected, YN144-infected, and the control groups. iTRAQ ratios (YN13/YN144/Control) are shown on the right.

To confirm the result of LC-MS/MS, five proteins (MX1, Annexin A4 (ANXA4), heat shock protein 90 kDa beta member 1 (HSP90B1), Aminopeptidase N (APN), and β-actin) were chosen for Western blot analysis. As shown in Figure 5, the ratios of these five proteins among the two virus-infected groups and control group were consistent with the data from LC-MS/MS analysis.

## 4. Discussion

In this study, host responses to two different PEDV strains were analyzed using comparative proteomics. YN13 is a pathogenic PEDV strain causing severe acute diarrhea in piglets, while YN144 is an attenuated strain causing no significantly clinical signs. A total of 269 and 301 proteins were significantly altered in the jejunum tissues of the piglets inoculated with YN13 and YN144, respectively. A comparative analysis of these DEPs was performed to explore the pathogen-host interaction in jejunums of PEDV-infected pigs and identify reasons for the difference between the two PEDV strains in pathogenicity.

### 4.1. Cellular Immune Response

Proteins encoded by some ISGs have been reported to display excellent antiviral activity [23]. In the present study, we identified several ISG proteins, including ISG15, 2′-5′ oligoadenylate synthetase 1 (OAS1), OAS2, OASL, Mx1, Mx2, Tripartite motif-containing protein 21 (TRIM21), interferon-induced protein with tetratricopeptide repeats 1 (IFIT1), and IFIT3. All these proteins, except for MX1 and TRIM21, were significantly upregulated in both YN13 and YN14 groups, and MX1 and TRIM21 were upregulated only in the YN13-infected group, indicating that these ISG proteins may participate in the process of resisting PEDV infection and the virulent strain YN13 could induce more kinds of ISG proteins than the attenuated strain YN144. It is well known that expression of ISGs depends on the induction of interferon (IFN). Previous studies have demonstrated that the N protein, the non-structure protein 1, 3c-like protease or papain-like protease of PEDV, inhibited the production of type-I IFN in vitro [24,25,26,27]. In addition, Cao et al. have proven in vitro that PDEV could inhibit the production of IFN-β in its infected porcine intestinal epithelial cells [28]. However, recently, Thavamathi et al. have verified that suckling pigs infected with PEDV have a higher and earlier increase in serum IFNα [29], which may explain the upregulation of those ISG proteins in the present study. It is also known that IFN regulates the production of ISGs by activating the JAK-STAT signaling pathway [30]. Transcription of ISGs requires the phosphorylation and nuclear transportation of signal transducer and activator of transcription proteins (STATs). There are seven STAT proteins in mammals [31]. Among them, STAT1 and STAT2 are the most important in the IFN signaling pathway [23]. In vitro studies have demonstrated that PEDV infection inhibited the interferon signaling by targeted degradation of STAT1 [20,32]. However, in this work, no significant change was found in STAT1 and STAT2 in the two PEDV infected groups. So, further investigations are needed to elucidate the upregulation of ISG proteins.

### 4.2. Cytoskeletal Response

Cytoskeletal changes are related to transcellular membrane trafficking, which is facilitated for viral replication. In the current analysis, α-tubulin and β-tubulin were upregulated and downregulated, respectively, in both the YN13-infected group and YN144-infected group, which was consistent with the findings of other authors, who reported that α-tubulin was upregulated during infection of some other coronaviruses such as transmissible gastroenteritis coronavirus (TGEV) and severe acute respiratory syndrome (SARS) [33,34]. Downregulation of β-tubulins in host cells was also found during the infection of porcine circovirus type 2 [35]. Since tubulin was involved in the process of coordinating viral entry, replication, assembly, and egress [36,37], the alteration of tubulin in this study might be a mechanism for PEDV to coordinate its life cycle in host cells.

### 4.3. Different Effects of Virulent and Attenuated Strains on Inducing Heat Shock Proteins 

Viruses depend on the host-chaperone machinery for their protein folding and viral assembly [38,39]. Interestingly, most HSPs identified in this study, including heat shock protein 90 kDa beta member 1 (HSP90B1), heat shock protein 90 kDa alpha, class B member 1 (HSP90AB1), heat shock 70 kDa protein 8 (HSPA8), heat shock protein 40 kDa subfamily A member 1 (DNAJA1) and heat shock protein 40 kDa subfamily C member 10 (DNAJC10), were significantly upregulated in the YN13-infected group, but were down-regulated in the YN144-infected group.

Hsp90 is a molecular chaperone that guides the folding, intracellular disposition, and proteolytic turnover of many key regulators of cell growth and differentiation [40]. Previous reports showed that HSP90 plays a role in the replication of both RNA viruses and DNA viruses [39], and inhibiting the expression of HSP90 could suppress the replication of many viruses such as human immunodeficiency virus, rotavirus, influenza virus, and porcine reproductive and respiratory syndrome virus [41,42,43,44]. This indicated HSP90 possessed a positive effect on the replication on these viruses.

HSP70, the most conserved HSP, is a multifunctional protein that plays a role in cellular signal transduction, regulation of cell cycle and cell death, and the folding status of proteins under stress conditions. It has been reported that HSP70 is involved in all phases of viral life cycles and plays an important role in regulating virus proliferation. The recruitment of HSP70 was thought to be a survival strategy for viruses in their hosts [45] and the positive effect of HSP70 on virus replication has been widely observed [46,47,48]. Quercetin, an inhibitor of the HSP70, has been reported to possess anti-PEDV ability [49]. However, since quercetin is not a special inhibitor of HSP70, the real function of HSP70 on PEDV is under investigation in our laboratory.

There are few reports describing the role of HSP40 in viral activities. However, since HSP40 participates in regulating the function of HSP70 [50], HSP40 may promote virus replication via regulating HSP70. Therefore, we speculate that YN13 may enhance its replication by upregulating the expression of HSP90B1, HSP90AB1, HSPA8, DNAJA1, and DNAJC10 while the downregulation of these five HSPs may inhibit the replication of YN144.

### 4.4. Different Effects of Virulent and Attenuated Strains on the Production and Translation of Subgenomic mRNA

Transcription and translation are two key steps in the process of virus replication. Alterations of factors involved in viral transcription and translation may affect viral replication. Coronavirus replication involves the generation of nested subgenomic mRNAs (sgmRNAs) with a common capped 5′ leader sequence [51]. To organize optical transcription of sgmRNAs, its 5′ terminal leader sequences need to interact with intergenic (IG) sequences just upstream of each open reading frame (ORF). Li et al. have verified that hnRNPA1, a RNA binding protein, can bind both the 5′ terminal leader sequence and IG sequence of coronavirus sgmRNA [52]. So, it is obvious that alteration of hnRNPA1 can influence the transcription of coronavirus and thus the replication of coronavirus. This phenomenon has been verified by a previous study reporting that overexpression of hnRNPA1 promoted mouse hepatitis virus (MHV) replication while a dominant-negative inhibition of hnRNPA1 reduced MHV replication [53]. In this study, hnRNPA1 was downregulated in the YN144-infected group, suggesting a decrease of the replication of YN144. The translation of most of the sgmRNAs requires eukaryotic initiation factor 4F (eIF4F), which is composed of eIF4A, eIF4E, and eIF4G. Regina Cencic et al. have reported that replication of human coronavirus 229E was significantly inhibited by blocking the interaction between eIF4E and eIF4G [51]. In the case of vesicular stomatitis virus and influenza virus, viral replication was also inhibited by silencing eIF4G1 [54,55]. In the present study, eIF4G1 was upregulated in YN13-infected group but downregulated in YN144-infected group. This result demonstrates that YN13 might enhance its replication by upregulating expressed eIF4G, while the downregulation of eIF4G1 may result in inhibiting the replication of YN144.

According to our analysis above, the difference between YN13 and YN144 in replication ability might be due to the difference between the two PEDV-infection groups in alterations of eIF4G1, hnRNPA1, HSP90B1, HSP90AB1, HSPA8, DNAJA1, and DNAJC10 in jejunums of pigs. Since virus pathogenicity is closely related to its replication in the host, we speculate that the difference between these two PEDV-infected groups in their changes of the proteins might be responsible for their difference in pathogenicity.

In summary, iTRAQ combined with LC-MS/MS was used to characterize the global proteomic changes in jejunums from pigs infected with a highly virulent PEDV strain YN13 and its attenuated PEDV strain YN144. To the best of our knowledge, this is the first quantitative proteomic work on the in vivo responses to PEDV infection. We discovered a new mechanism of host against PEDV and identified relevant targets for further studies of PEDV pathogenesis, which might provide novel insights into the prevention of PEDV infection.

## Supplementary Materials

The following are available online at www.mdpi.com/1999-4915/8/12/323/s1. Figure S1: YN13 caused diarrhea of piglets in YN13-infected group; Table S1: Villus height/crypt depth (V/C) of piglet’s duodenum, jejunum, and ileum; Figure S2: Villus height/crypt depth (V/C) of piglet’s duodenum, jejunum, and ileum. File S1: Information for all significantly altered proteins identified in YN13 and YN144-infected groups; File S2: Gene Ontology analysis of significantly differently expressed proteins in YN13 and YN144-infected groups; File S3: Functional characterization of significantly differently expressed proteins in YN13 and YN144-infected groups.

## Abbreviations

ANXA4	Annexin A4
APN	Aminopeptidase N
DEPs	differently expressed proteins
DNAJ	heat shock protein 40 KD
dpi	days post infection
eIF	eukaryotic initiation factor
hnRNPA1	heterogeneous nuclear ribonucleoprotein A1
IFN	interferon
IPA	*ingenuity pathway analysis*
ISG	interferon-stimulated gene
MHV	mouse hepatitis virus
sgmRNAs	subgenomic mRNAs
PEDV	porcine epidemic diarrhea virus
TGEV	transmissible gastroenteritis virus
