# Supplementary material for: Comparative Proteome Analysis of Porcine Jejunum Tissues in Response to a Virulent Strain of Porcine Epidemic Diarrhea Virus and Its Attenuated Strain
Source: Viruses. 2016 Nov 29;8(12):323. doi: 10.3390/v8120323 (PMC5192384; doi:10.3390/v8120323)
Supplement: Supplementary file 1 [file viruses-08-00323-s001.zip › viruses-147569-supplementary-final/Figure S1, Figure S2 , Figure S3 and Table S1.docx]

**Comparative Proteome Analysis of Porcine Jejunum Tissues in Response to a Virulent Strain of Porcine Epidemic Diarrhea Virus and Its Attenuated Strain**

**Zhonghua Li, Fangzhou Chen, Shiyi Ye, Xiaozhen Guo, Atta Muhanmmad Memon,
Meizhou Wu and Qigai He ***


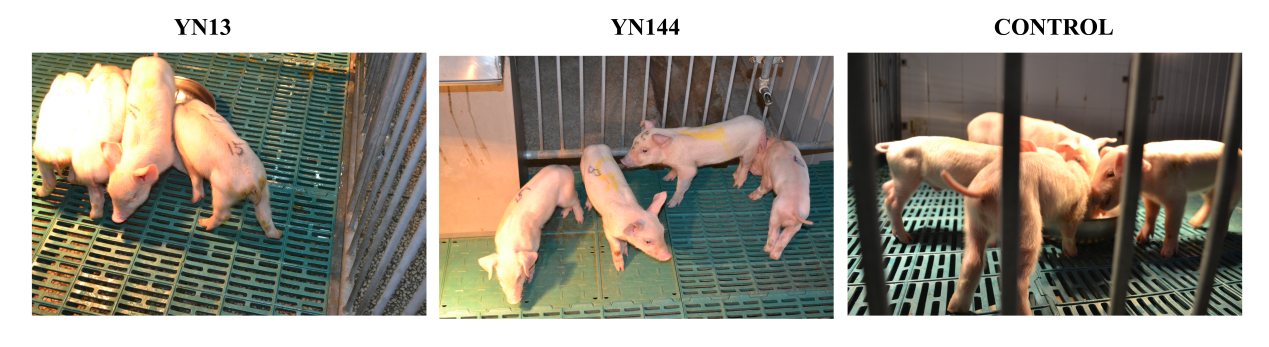
**Figure S1**. YN13 caused diarrhea of piglets in YN13-infected group.

**Table S1**. Villus height/crypt depth (V/C) of piglet’s duodenum, jejunum, and ileum. Two sections were selected for each sample. The three longest villi and [deepest](javascript:void(0);) crypts were measured for each section. The dates were shown in the form of mean±[standard](javascript:void(0);) [deviation](javascript:void(0);).

|  | **Group** | **Villus length (μm)** | **Crypt depth (μm)** | **V/C** |
| --- | --- | --- | --- | --- |
| **Duodenum** | YN13 | 592.44±30.35 | 167.49±2.26 | 3.58±0.21 |
|  | YN144 | 628.80±5.15 | 170.43±2.68 | 3.69±0.05 |
|  | Control | 651.70±19.91 | 163.08±6.90 | 4.00±0.16 |
| **Jejunum** | YN13 | 468.53±43.05 | 185.27±7.37 | 2.52±0.13 |
|  | YN144 | 553.15±32.35 | 156.06±9.22 | 3.54±0.11 |
|  | Control | 600.82±13.15 | 151.73±9.12 | 3.97±0.20 |
| **Ileum** | YN13 | 445.40±9.92 | 153.60±14.40 | 2.92±0.21 |
|  | YN144 | 522.83±13.79 | 142.24±4.22 | 3.89±0.08 |
|  | Control | 570.54±12.91 | 138.53±2.46 | 4.12±0.15 |


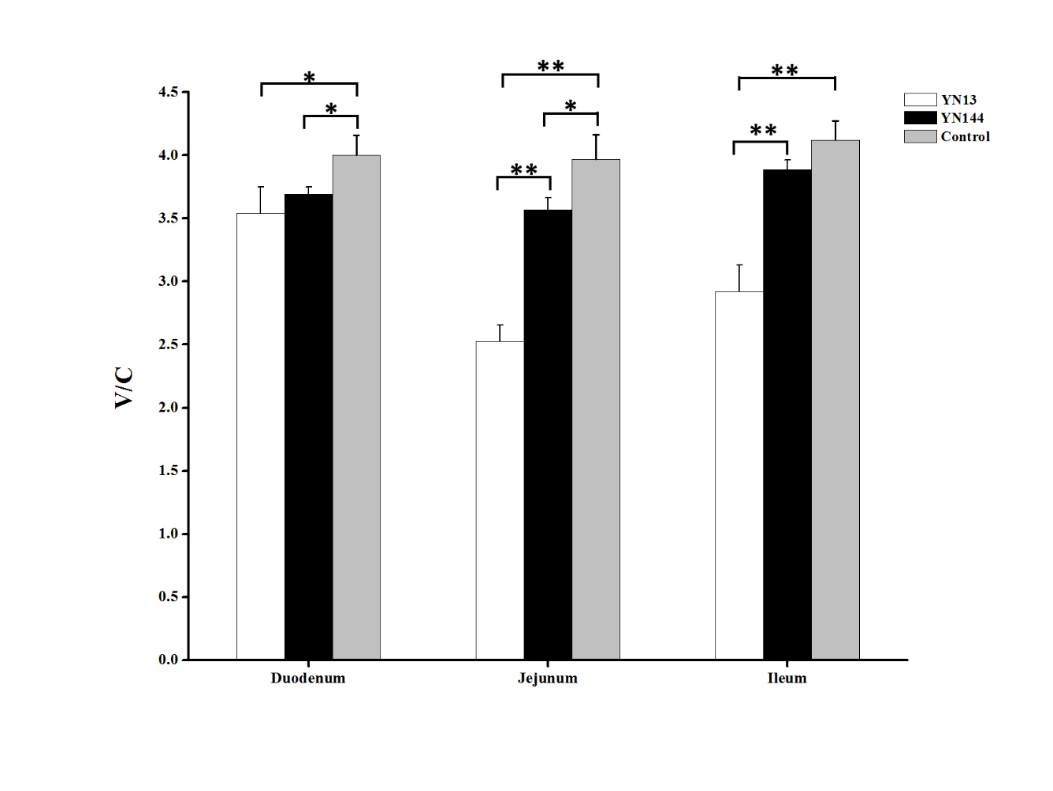


**Figure S2**. Villus height/crypt depth (V/C) of piglet’s duodenum, jejunum, and ileum. Differences between the two groups were determined using an independent sample t test. *0.01<p<0.05, ** p < 0.01.


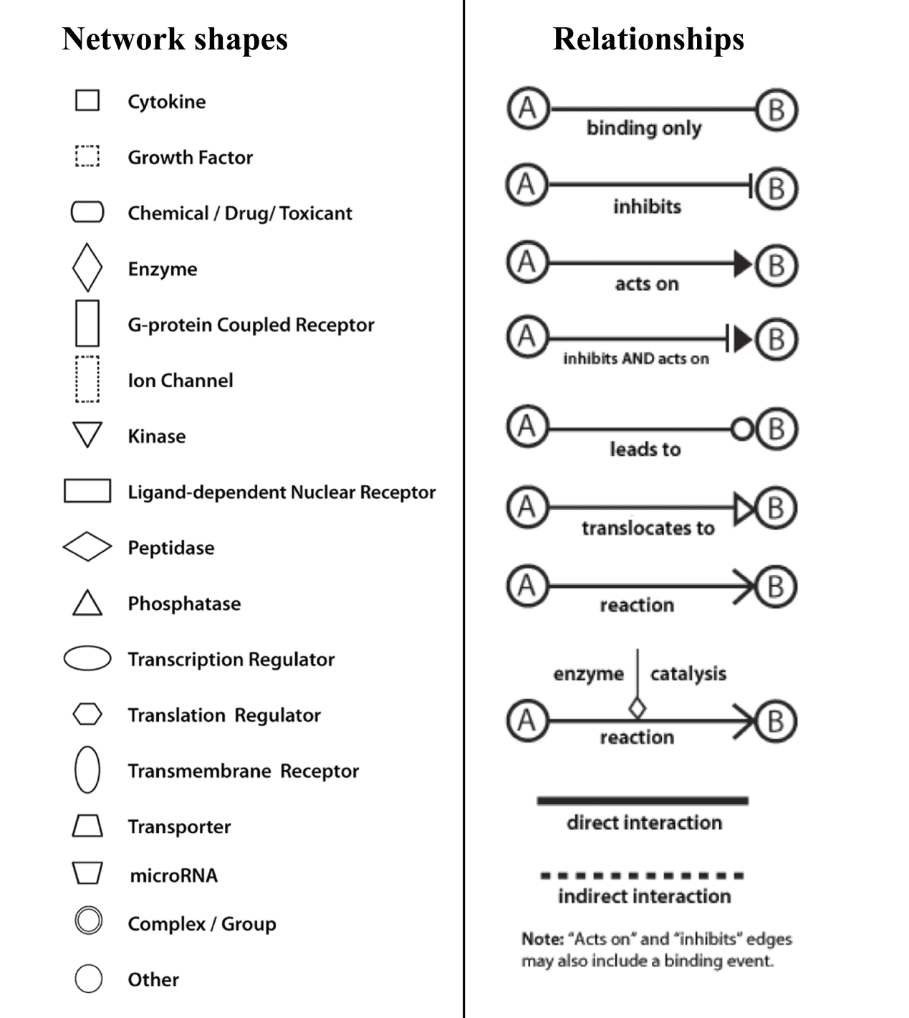


**Figure S3**. Explanations for the symbols in the specific network analysis.

© 2016 by the authors; licensee MDPI, Basel, Switzerland. This article is an open access article distributed under the terms and conditions of the Creative Commons by Attribution (CC-BY) license (http://creativecommons.org/licenses/by/4.0/).
